# Altered individual-level morphological similarity network in children with growth hormone deficiency

**DOI:** 10.1186/s11689-024-09566-5

**Published:** 2024-08-26

**Authors:** Yanglei Cheng, Liping Lin, Weifeng Hou, Huaqiong Qiu, Chengfen Deng, Zi Yan, Long Qian, Wei Cui, Yanbing Li, Zhiyun Yang, Qiuli Chen, Shu Su

**Affiliations:** 1 Department of Radiology, The First Affiliated Hospital, Sun Yat-sen University, Guangzhou, China; 2 Department of Endocrine, The First Affiliated Hospital, Sun Yat-sen University, Guangzhou, China; 3 Department of Pediatric, The First Affiliated Hospital, Sun Yat-sen University, Guangzhou, China; 4 Department of Biomedical Engineering, College of Engineering, Peking University, Beijing, China

**Keywords:** Structural MRI, Morphological brain networks, Topological organization, Growth hormone deficiency

## Abstract

**Background:**

Accumulating evidences indicate regional grey matter (GM) morphology alterations in pediatric growth hormone deficiency (GHD); however, large-scale morphological brain networks (MBNs) undergo these patients remains unclear.

**Objective:**

To investigate the topological organization of individual-level MBNs in pediatric GHD.

**Methods:**

Sixty-one GHD and 42 typically developing controls (TDs) were enrolled. Inter-regional morphological similarity of GM was taken to construct individual-level MBNs. Between-group differences of topological parameters and network-based statistics analysis were compared. Finally, association relationship between network properties and clinical variables was analyzed.

**Results:**

Compared to TDs, GHD indicated a disturbance in the normal small-world organization, reflected by increased L_p_, γ, λ, σ and decreased C_p_, E_glob_ (all *P*_*FDR*_ < 0.017). Regarding nodal properties, GHD exhibited increased nodal profiles at cerebellum 4-5, central executive network-related left inferior frontal gyrus, limbic regions-related right posterior cingulate gyrus, left hippocampus, and bilateral pallidum, thalamus (all *P*_*FDR*_ < 0.05). Meanwhile, GHD exhibited decreased nodal profiles at sensorimotor network -related bilateral paracentral lobule, default-mode network-related left superior frontal gyrus, visual network -related right lingual gyrus, auditory network-related right superior temporal gyrus and bilateral amygdala, right cerebellum 3, bilateral cerebellum 10, vermis 1-2, 3, 4-5, 6 (all *P*_*FDR*_ < 0.05). Furthermore, serum markers and behavior scores in GHD group were correlated with altered nodal profiles (*P* ≤ 0.046, uncorrected).

**Conclusion:**

GHD undergo an extensive reorganization in large-scale individual-level MBNs, probably due to abnormal cortico-striatal-thalamo-cerebellum loops, cortico-limbic-cerebellum, dorsal visual-sensorimotor-striatal, and auditory-cerebellum circuitry. This study highlights the crucial role of abnormal morphological connectivity underlying GHD, which might result in their relatively slower development in motor, cognitive, and linguistic functional within behavior problem performance.

**Supplementary Information:**

The online version contains supplementary material available at 10.1186/s11689-024-09566-5.

## Key points

1. GHD children undergo an extensive and significant reorganization in large-scale individual-level MBNs.

2. Abnormal morphological connectivity was found in cortico-striatal-thalamo-cerebellum loops, cortico-limbic-cerebellum, dorsal visual-sensorimotor-striatal, and auditory-cerebellum circuitry underlying pediatric GHD.

**Supplementary Information:**

The online version contains supplementary material available at 10.1186/s11689-024-09566-5.

## Introduction

Growth hormone deficiency (GHD) is the most common type of pathological short stature, which characterized by the decreased secretion of GH from the anterior pituitary [1]. GH can stimulate insulin-like growth factor-1 (IGF-1) secretion in liver [2], and GH/IGF-1 axis plays pivotal roles in linear growth, energy homeostasis, and cognitive function [2]. Specifically, GH receptors and IGF-1 receptors are expressed throughout human brain, including amygdala, hippocampus, and parahippocampal areas [3].Besides, IGF-1 signaling is critical in reducing apoptosis, promoting synaptic plasticity and long-term proliferation of neural stem cells, and plays a crucial part in early brain development, neurogenesis, and brain remodeling [4]. These neurogenic processes directly influence the development of brain structures, which in turn affect various brain functions. The important roles of GH/IGF-1 axis in nervous system development, function, and metabolism have been extensively demonstrated [5, 6]. However, the roles of GH deficiency in nervous system development from a large-scale morphological brain network organization were not yet illustrated.

Nowadays, structural neuroimaging has revealed that children with GHD showed reduced thalamic, globus pallidum, and hippocampus volumes that were related to deficits in cognitive function and motor performance [7]. It was also reported that morphological changes in the cerebral cortex in children with isolated GHD, mainly distributed around the bilateral central sulci and the lateral and basal parts of the temporal lobes, which were partially influenced by GH and IGF-I levels [8, 9]. In addition, in young adult male patients with childhood-onset GH deficiency have alterations in cortical thickness in different brain lobes/regions [10]. These above results [7–10] provide numerous structural evidences for macro morphological abnormalities in GHD from childhood to adulthood. However, the human brain is an extremely complex system in which neurons, clusters of neurons, or regions are connected to form a complex network [11, 12], it is essential to consider the role of regional volumes alterations in the context of the whole brain network topology.

Previous studies based on resting-state functional magnetic resonance imaging (rs-fMRI) have revealed brain network dysfunctions underlying GHD from the aspects of functional connectivity density [13] and dynamic brain network [14]. However, functional brain networks are characterized by synchronized brain activity at a certain point in time, while structural networks reflect more stable patterns of the anatomical organization affected by physiological hormones, heredity, experience-related plasticity, and mutually trophic reinforcement [15, 16]. Morphological brain networks (MBNs) based on structural MRI have become essential for studying human brain connectomes. A cortical feature-based structural connectivity network can locate specific altered cortical regions and indicate how their connectivity and functions may change. Moreover, individual-based MBNs can reflect synchronous maturation intensities between anatomical regions at the individual level during brain development [17, 18]. Over recent years, individual-based MBNs of grey matter (GM) have become increasingly popular [19–22], however, the organization of MBNs in children with GHD are not fully estimated. Hence, understanding the morphological abnormalities and large-scale network alterations is critical to reveal the pathophysiology and neurodevelopment mechanisms underlying pediatric GHD.

In current study, we compute individual-based GM networks based on the inter-regional morphological similarities of GM [23], and further examine whether the clinical variables of pediatric GHD are associated with significant brain GM network properties.

## Materials and methods

### Participants and clinical assessment

**Fig. 1:**
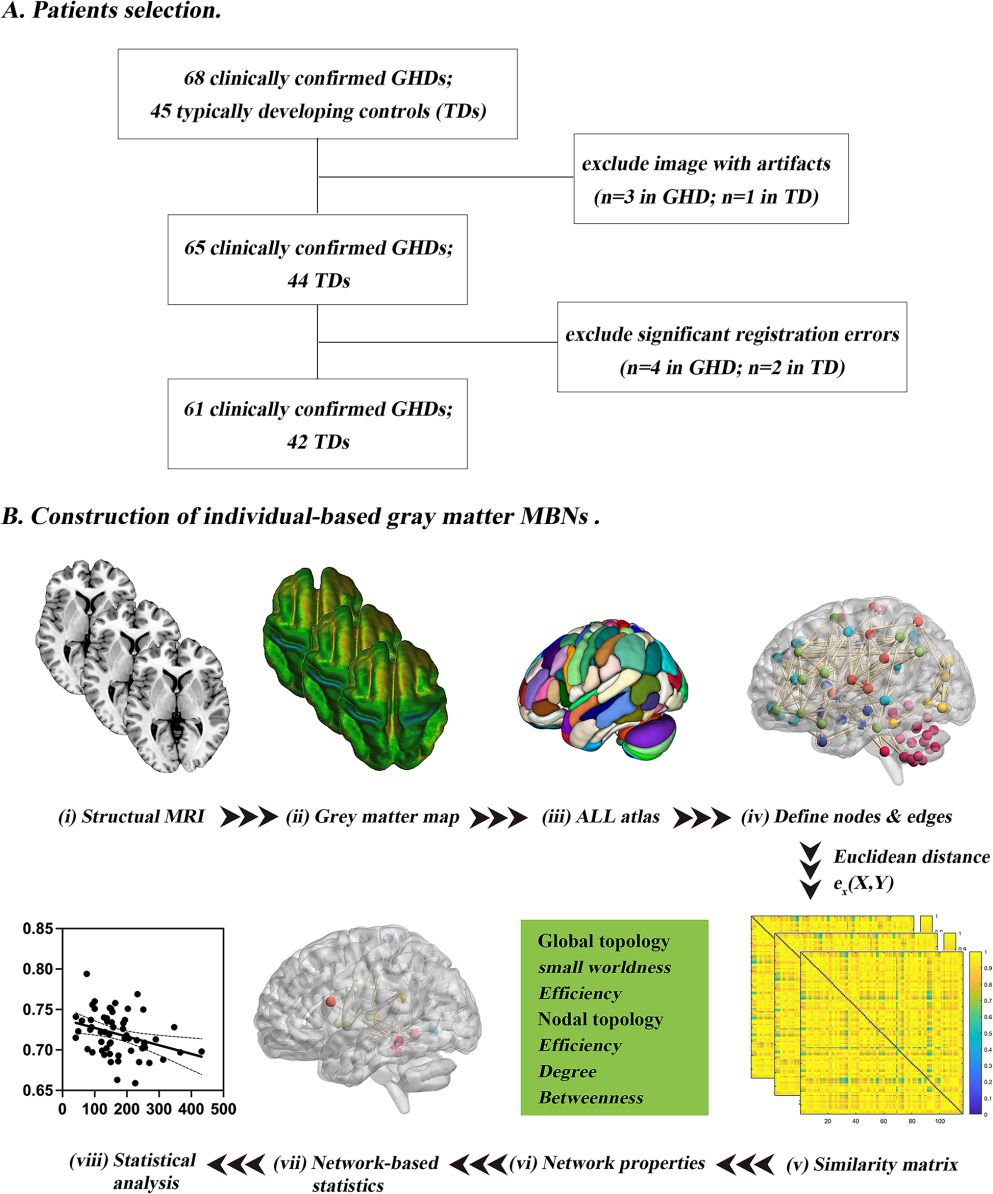
Flowchart of selection of GHD group (**A**) and the primary analytical process of gray matter MBNs (**B**) in the current study. (i) 3D T1-weighted imaging and (ii) preprocessing (segment, normalize, modulate, and smooth); (iii) nodes are defined according to the automated anatomical labelling (AAL-116) atlas; (iv) edges are defined according to the combined Euclidean distance method; (v) an individual similarity matrix is obtained; (vi-vii) network properties are calculated and analyzed

This study protocol was approved by the ethics committees of our institution (No.2021082) and registered at https://clinicaltrials.gov/ (Identifier: ChiCTR2100048109). Written informed consent was obtained from all participants’ guardians. From November 2020 to June 2023, 68 pediatric GHD were prospectively recruited. Meanwhile, 45 typically developing controls (TDs) matched for age and gender was recruited. Detailed clinical and demographic data of the participants are shown in Table 1. Finally, 61 pediatric GHD were enrolled, and 7 patients were excluded due to image artifacts (*n* = 3) and significant registration errors (*n* = 4), three TDs were excluded due to image artifacts (*n* = 1) and significant registration errors (*n* = 2) (Fig. 1A).

**Table 1:** Demographic and clinical characteristics of children with GHD and HCs

**Characteristics**	**GHD (*n* =61**)	**TD (*n* =42**)	***P* value**
**Age, years**	8.93 ± 2.86	8.95 ± 2.25	0.965
**Male/Female(n)**	40/21	28/14	0.908
**Height (cm)**	121.34 ± 0.15	134.24 ± 11.33	< 0.001^*^
**Height SDS**	-2.33 ± 0.44	0.34 ± 0.027	< 0.001^*^
**Weight (kg)**	23.59 ± 7.70	29.86 ± 7.03	< 0.001^*^
**Weight SDS**	-1.69 ± 0.77	0.23 ± 0.089	< 0.001^*^
**Body mass index (kg/m**^**2**^**)**	15. 62 ± 2.30	16.17 ± 1.68	0.563
**IGF-1 (ng/ml)**	171. 89 ± 80.71	NA	NA
**Peak GH level (μg/l)**	3.14 ± 1.57	NA	NA
**ACTH (pmol/L)**	6.02 ± 3.27	NA	NA
**Cortisol (μg/dl)**	10.32 ± 3.09	NA	NA
**TSH (μIU/mL)**	3.23 ± 3.34	NA	NA
**Total scores of Achenbach's CBCL**	43.58 ± 24.66	NA	NA

Data are represented as the mean ± SD. For comparisons of demographics, *P*-values are obtained using two sample t-test or Chi-square test; *P < 0.05 was considered significant

*GHD* growth hormone deficiency, *TD* typically developing, *SDS* standard deviation scores, *IGF-1* Insulin-like growth factor -1, *GH* growth hormone, *ACTH* adreno-cortico-tropic-hormone, *TSH* thyroid stimulating hormone, *Achenbach's CBCL* Achenbach's child behavior cheek list

For children with GHD, age, gender, height, weight, body mass index (BMI), serum IGF-1, adrenocorticotropic hormone (ACTH), cortisol, and thyroid stimulating hormone (TSH) were obtained from the medical records. Furthermore, GHD children underwent two provocation tests. Blood samples were collected at time 0 and after 30, 60, 90, 120 minutes after intravenous bolus injection of pyridostigmine combined with levodopa. The GH peak was recorded after the provocation tests. The Achenbach's child behavior cheek list (CBCL) was also assigned. Regarding TDs, age, gender, height, weight, and BMI were also recorded.

The inclusion criteria of pediatric GHD: 1) short stature, less than the third percentile or below 2 standard deviations of mean age-matched population height; 2) less than 10 μg/L peak serum GH level with at least two provocative stimulations; 3) no adrenocorticotropic hormone deficiency, hypoglycemia, thyroid-related diseases, and familial genetic and metabolic diseases; 4) right-handedness. The exclusion criteria of each participant were: 1) combined with other mental disorders, personality disorders, or psychotropic drug dependence; 2) challenging to cooperate during MRI examination, and the image quality is too poor for image analysis; 3) a history of other brains organic and metabolic diseases; 4) other MRI contraindications.

### Image acquisition and preprocessing

All participants underwent sagittal three-dimensional T1 imaging with a 3.0-T MR imaging system (SIGNA Pioneer GE Healthcare, WI, USA) using a 32-channel phased-array head coil. The head was stabilized with cushions and earplugs. Images were acquired using the fast spoiled gradient recalled echo (FSPGR) sequence, with the following parameters: repetition time (TR) = 8.6 ms, echo time (TE) = 3.3 ms, flip angle = 12°, 188 sagittal slices with slice thickness = 1 mm with no slice gap, a field of view= 256 × 256 mm^2^, and data matrix = 256 × 256.

Automated segmentation of the whole brain based on 3D T1-weighted images was processed with the CAT12 toolbox (http://www.neuro.uni-jena.de/cat/) within the SPM12 environment (http://www.fil.ion.ucl.ac.uk/spm/software/spm12/) running under MATLAB R2019b (MathWorks). The preprocessing steps involved spatial normalization to the Montreal Neurologic Institute (MNI) space and segmentation. Modulated GM images were resliced to a 2 mm isotropic voxel size and spatially smoothed using a 3D Gaussian kernel with an FWHM of 6 mm, which was chosen in line with previous studies [24, 21]. T1-weighted were downsampled from the raw 1 mm to 2 mm isotropic voxel size, consistent with previous individual-level morphological brain network studies [21, 24]. This resolution balances anatomical detail preservation with computational efficiency for whole-brain network analysis.

### Construction of individual morphological similarity networks 

The Anatomical Automatic Labeling (AAL-116) atlas [25] was applied to define network nodes or brain regions, and each hemisphere was divided into 45 anatomical regions of interest (ROIs) and cerebellum was divided into 26 anatomical ROIs. Next, the approach named Multivariate Euclidean Distances (MEDs) [23] was performed to estimate the inter-regional morphological similarities between each of the 6670 pairs of the 116 cortical, subcortical and cerebellum regions derived from each individual GMV Map. We calculated Euclidean Distances based on gray matter volumetric values rather than spatial coordinates. This approach assumes that regions with similar gray matter volumes across subjects are more likely to be structurally connected. The details of MEDs were previously described [23], and we outline the key aspects of this algorithm here for clarity and completeness:

- Step A) Compute the inter-regional combined Euclidean distance

For the $k$ th subject, each pair of anatomical regions $(X,Y)$ from the AAL template was computed using the combined Euclidean distance ${e}_{k}(X,Y)$, defined as follows:

1 $$e_k\left(X,Y\right)=\frac{n1n2}{n1+n2}\left(\frac2{n1n2}\sum\nolimits_{i=1}^{n1}\sum\nolimits_{j=1}^{n2}{\Arrowvert x_i-y_i\Arrowvert}_2-\frac1{n_1^2}\sum\nolimits_{i=1}^{n1}\sum\nolimits_{j=1}^{n1}{\Arrowvert x_i-x_j\Arrowvert}_2-\frac1{n_2^2}\sum\nolimits_{i=1}^{n2}\sum\nolimits_{j=1}^{n2}{\Arrowvert y_i-y_j\Arrowvert}_2\right)$$

Here $X=\{{x}_{1}, \dots , {x}_{n1}\}$ and $Y=\{{y}_{1}, \dots , {y}_{n2}\}$, where $x$ and $y$ denote vertices in regions $X$ and $Y$, respectively. ${n}_{1}$ and ${n}_{2}$ are the numbers of vertices in $X$ and $Y$. The Euclidean distance is computed by the 2-norm (${\parallel .\parallel }_{2}$).

- Step B) Perform Min- Max normalization

The Min-Max normalization was performed to minimize possible bias in different ranges of different subjects. The Min-Max normalization between regions X and Y of the $k$ th subject is computed as follows:

2 $${e}_{k\_n}\left(X,Y\right)=\frac{{e}_{k}\left(X,Y\right)-{e}_{k\_min}}{{e}_{k\_max}-{e}_{k\_min}}$$

Where ${e}_{k\_min}$ and ${e}_{k\_max}$ are the minimum and maximal value in the combined Euclidean distance of the $k$ th subject, respectively.

- Step C) Define the similarity measurement

In the last step, to obtain the morphological similarity, the value of ${e}_{k\_n}\left(X,Y\right)$ should be converted to a similarity measurement using the following equation;

3 $${c}_{k}\left(X,Y\right)=\text{exp}(-{e}_{k\_n}\left(X,Y\right))$$

Finally, a 116×116 MBNs of each subject was obtained. The values of the edges range from 0 to 1, and 1 represents identical morphological feature distributions in the two AAL regions. A flowchart of the construction of individual-level grey matter MBNs is presented in Fig. 1B.

### Network analysis

Network properties were calculated using GRETNA toolbox (http://www.nitrc.org/projects/gretna/) [26] in MATLAB. To ensure the thresholder networks were estimable with sparse properties and small-world index was > 1.0 [27], the minimum and maximum sparsity values were determined. Then, the threshold range was set as 0.05 < S < 0.40 with an interval of 0.05. At each sparsity level, the topologic profiles of brain networks at both global and nodal levels were calculated. Global network profiles included the clustering coefficient (C_p_), characteristic path length (L_p_), normalized clustering coefficient (γ), normalized characteristic path length (λ), small-world parameters (σ), global efficiency (E_glob_), local efficiency (E_loc_), and nodal network topological profiles including nodal efficiency (${E}_{i}$), nodal degree (${D}_{i}$), and nodal betweenness (${B}_{i}$). Considering the network sparsity dependent network characteristics, the area under the curve (AUC) of each global profile (E_glob_, E_loc_, C_p_, L_p_, $\gamma$, $\lambda$, $\sigma$) and nodal profile (${B}_{i}$, ${E}_{i}$, ${D}_{i}$) across a range of interested densities were calculated as the summarized scalar for each measure, denoted as ${E}_{glob}^{auc}$, ${E}_{loc}^{auc}$, ${C}_{P}^{auc}$, ${L}_{P}^{auc}$, ${\gamma }^{auc}$, ${\lambda }^{auc}$, ${\sigma }^{auc}$, ${B}_{i}^{auc}$, ${E}_{i}^{auc}$, ${D}_{i}^{auc}$, respectively.

### Network-based statistics analysis

To identify the differences in brain network connectivity between GHD and TD groups, the network-based statistics (NBS) method [28] was also used. The NBS has become increasingly popular in recent years for network-level statistical analyses in neuroimaging studies [21, 24], as it offers greater sensitivity in detecting connected components of altered connectivity compared to traditional mass-univariate approaches. To perform NBS analysis, in current study, we first examined whole-brain networks to identify nodes showing significant between-group differences (*P* < 0.05, uncorrected) in at least one centrality measure (degree, efficiency, or betweenness), which resulted in a set of suprathreshold connections. Within the set of suprathreshold connections, we identified topologically connected components using a breadth-first search algorithm [28]. The size of each identified component was compared against a null distribution obtained through permutation testing (5000 permutations). This step controlled for multiple comparisons at the component level. Significant components (*P*_*FDR*_ < 0.05) were visualized and interpreted as subnetworks showing between-group differences in connectivity [28].

### Statistical analysis

SPSS v21.0 (IBM Corp., Armonk, New York) was used to perform statistical analysis. The Shapiro-Wilk test was used to evaluate the normality of the data for continuous variables. Qualitative variables were compared by Chi-squared tests, and quantitative variables were compared using two-tailed independent-sample t-tests. A *P* < 0.05 was set as statistically significant.

Analysis of covariance (ANCOVA) was used to compare between-group differences of the AUC of each network metric (including global and nodal metrics) with diagnosis as fixed factors, age, and gender added to the model as covariates, respectively. The Benjamini-Hochberg false discovery rate (BHFDR) correction was applied to multiple comparisons. Finally, partial correlation analysis was used to examine relationships between significant network metrics and clinical variables, controlling for age and gender as confounding variables (*P* < 0.05).

### Reproducibility analyses

Similar network analysis was repeated for reproducibility analysis with an additional Harvard Oxford atlas with 112 brain regions (HOA-112 atlas, Table S1) [29] to evaluate the potential effects of different parcellation schemes.

## Results

### Demographic information and clinical characteristics

Finally, sixty-one GHD (40 male; mean age, 8.93 ± 2.86 years) and 42 TDs (28 male; mean age, 8.95 ± 2.25 years) were enrolled. The detailed demographic and clinical characteristics were shown in Table 1. The values of ACTH, cortisol, and TSH in the children with GHD were within the normal range standardized for age and sex.

### Between-group differences in global properties of GM networks

**Fig. 2:**
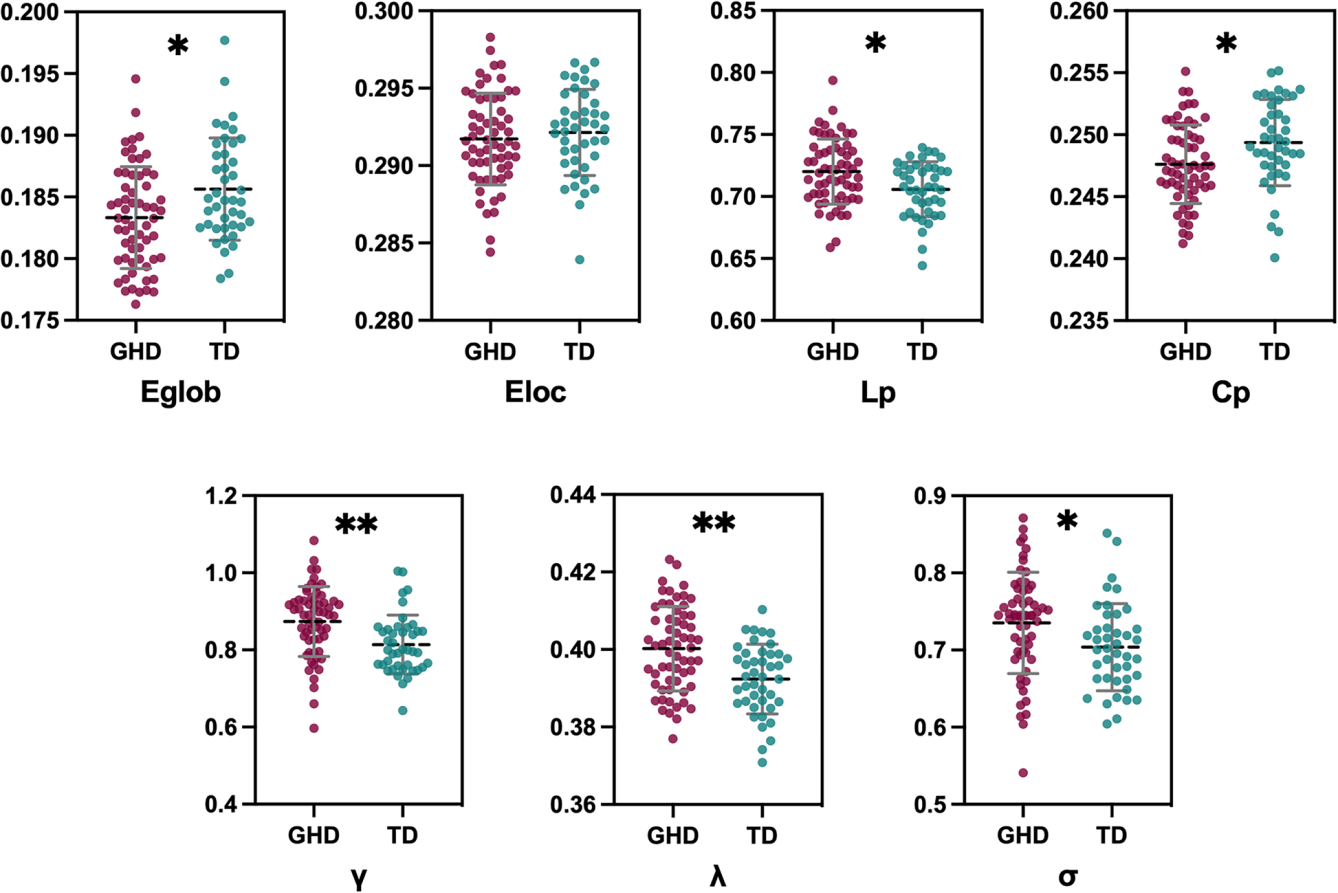
Comparison of global parameters of the brain anatomical networks between the GHD group and typically developing (TD) controls. Abbreviations: GHD = growth hormone deficiency; TD = typically developing; E_glob_ = global efficiency; E_loc_ = local efficiency; C_p_ = clustering coefficient; L_p_ = shortest path length; λ = normalized characteristic path length; γ = normalized clustering coefficient; δ = λ/γ, small-world characteristic. Error bars represent the standard deviation of the mean. ^*^P < 0.05, ^**^P < 0.001, compared with TDs

Within the defined threshold range, GHD and TDs exhibited normalized C_p_ values (greater than 1) and L_p_ values (approximately equal to 1), which showed typical features of small-world architecture in GM morphological networks. Compared with TDs, GHD children exhibited significantly decreased E_glob_ (F = 9.31, *P* = 0.003), C_p_ (F = 7.23, *P* = 0.008) and increased L_p_ (F = 10.08, *P* = 0.002), γ (F = 12.45, *P* < 0.001), λ (F = 18.10, *P* < 0.001) σ (F = 6.21, *P* = 0.014) values (Fig. 2). However, no significant difference was observed in E_loc_ (*P* = 0.465) between group comparisons (Fig. 2).

### Between-group differences in nodal profiles of GM networks

**Fig. 3:**
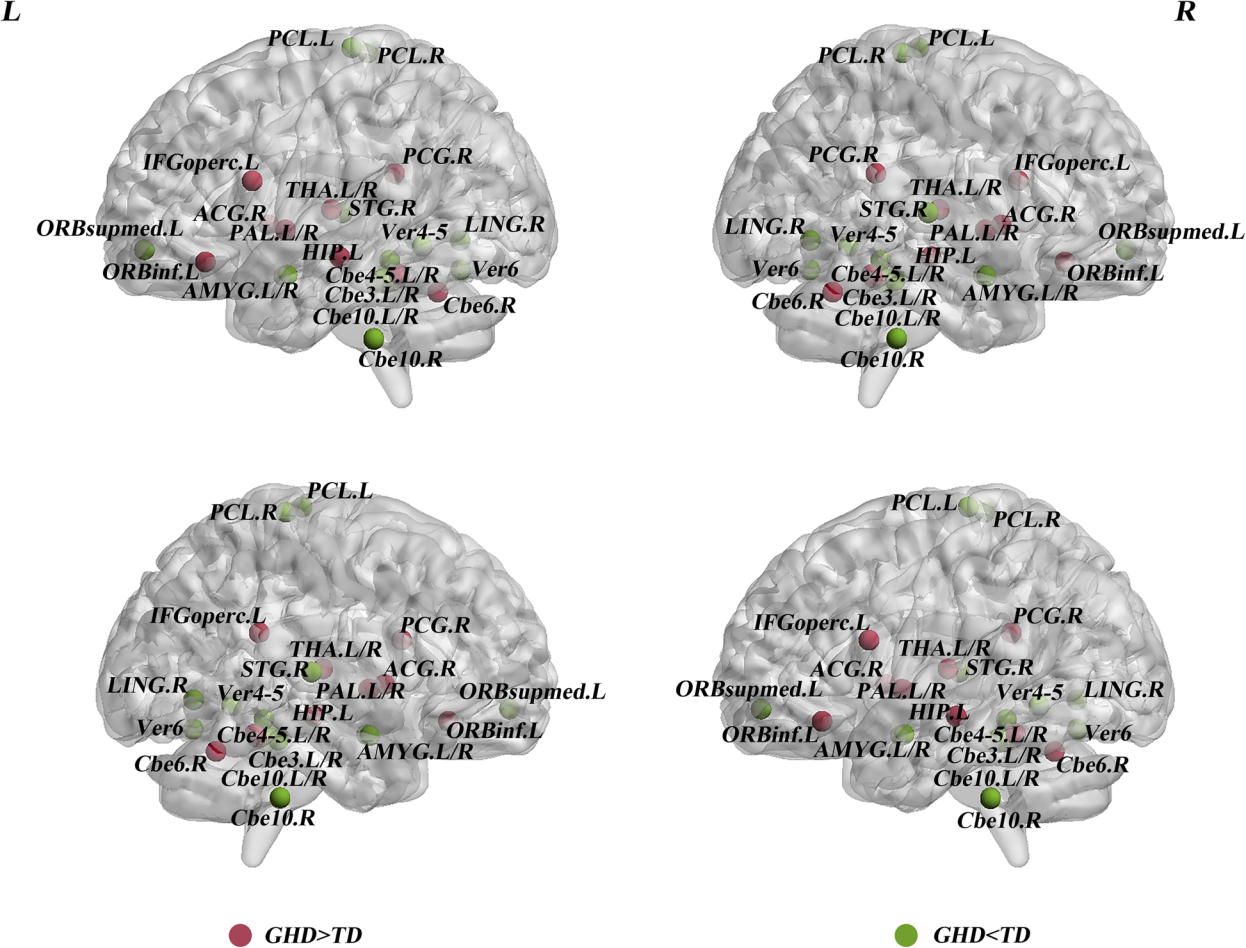
Compared to TDs, GHD group showed regions of altered nodal profiles, showing increased points (red) and decreased (green) points. The detailed information can be found in Table 2. Abbreviations: GHD = growth hormone deficiency; TD = typically developing; IFGoperc = Inferior frontal gyrus, opercular part; ORBinf = Inferior frontal gyrus, orbital part; ACG = Anterior cingulate and paracingulate gyri; PCG = Posterior cingulate gyrus; HIP = Hippocampus; PAL = Lenticular nucleus, pallidum; THA = Thalamus; Cbe4-5 = Cerebellum 4-5; Cbe6 = Cerebellum 6; ORBsupmed = Superior frontal gyrus, medial orbital; AMYG = Amygdala; LING = Lingual gyrus; PCL = Paracentral lobule; STG= Superior temporal gyrus; Cbe3 = Cerebellum 3; Cbe10 = Cerebellum 10; Ver1-2 = Vermis 1-2; Ver3 = Vermis 3; Ver4-5 = Vermis 4-5; Ver6 = Vermis 6; L = left; R = right.

Compared to TDs, GHD children showed altered nodal profiles in widespread regions (*P* < 0.05, uncorrected), four were in the subcortical cortex, four were in the prefrontal cortex, four were in the temporal cortex, one was in the occipital cortex, three were in the parietal cortex, and eleven were in the cerebellum cortex (Fig. 3;Table 2). Specifically, in comparison with TDs, GHD exhibited significantly increased nodal profiles in the left inferior frontal gyrus, orbital part (ORBinf.L), limbic regions [right posterior cingulate gyrus (PCG.R) and left hippocampus], cerebellum region (right cerebellum 4-5) and bilateral pallidum, thalamus (*P*_*FDR*_ < 0.05; Fig. 3;Table 2); as well as significantly decreased nodal profiles in left superior frontal gyrus, medial orbital (ORBsupmed.L), right lingual gyrus (LING.R), right superior temporal gyrus (STG.R), bilateral paracentral lobule (PCL), limbic regions (bilateral amygdala), cerebellum regions (right cerebellum 3, bilateral cerebellum10 and Vermis 1-2, 3, 4-5, 6) (*P*_*FDR*_ < 0.05; Fig. 3;Table 2).

**Table 2:** Altered nodal profiles in GHD and typically developing controls

**Brain regions**	**Category**	***P*-value**		
		${D}_{i}^{auc}$	${E}_{i}^{auc}$	${B}_{i}^{auc}$
**GHD > TDs**				
IFGoperc.L	CEN	0.033^*^	0.439	0.007^*^
ORBinf.L	CEN	0.010^**^	0.011^*^	0.862
ACG.R	Limbic	0.009^*^	0.044^*^	0.804
PCG.R	Limbic	0.047^*^	0.607	0.002^**^
HIP.L	Limbic	0.088	0.455	0.022^**^
PAL.L	Striatum	0.064	0.257	<0.001^**^
PAL.R	Striatum	0.001^**^	0.009^*^	0.004^*^
THA.L	Thalamus	0.005^**^	0.032^*^	0.823
THA.R	Thalamus	0.005^**^	0.019^**^	0.167
Cbe4-5.L	Cerebellum	0.014^*^	0.034^*^	0.365
Cbe4-5.R	Cerebellum	0.001^**^	0.010^**^	0.420
Cbe6.R	Cerebellum	0.005^*^	0.032^*^	0.614
**GHD < TDs**				
ORBsupmed.L	DMN	0.008^*^	0.015^**^	0.021^*^
AMYG.L	Limbic	0.006^*^	0.034^**^	0.015^*^
AMYG.R	Limbic	0.005^**^	0.001^**^	0.192
LING.R	VN	0.042^*^	0.034^**^	0.846
PCL.L	SMN	0.016^*^	0.001^**^	0.433
PCL.R	SMN	0.104	0.034^**^	0.251
STG.R	AN	0.120	0.034^**^	0.257
Cbe3.L	Cerebellum	0.027^*^	0.015^*^	0.686
Cbe3.R	Cerebellum	<0.001^**^	<0.001^**^	0.009^*^
Cbe10.L	Cerebellum	0.614	0.034^**^	0.348
Cbe10.R	Cerebellum	0.404	0.021^**^	0.153
Ver1-2	Cerebellum	<0.001^**^	<0.001^**^	0.003^**^
Ver3	Cerebellum	<0.001^**^	<0.001^**^	<0.001^**^
Ver4-5	Cerebellum	0.021^**^	0.001^**^	0.234
Ver6	Cerebellum	0.006^*^	0.021^**^	0.561

27 regions with *P*-value <0.05 in at least one node profile were included.

*Abbreviations*:${D}_{i}^{auc}$ nodal degree, ${E}_{i}^{auc}$ nodal efficiency, ${B}_{i}^{auc}$ nodal betweenness, *GHD* growth hormone deficiency, *TDs* typically developing controls, *L* left, *R* right, *IFGoperc* Inferior frontal gyrus, opercular part, *ORBinf* Inferior frontal gyrus, orbital part, *ACG* Anterior cingulate and paracingulate gyri, *PCG* Posterior cingulate gyrus, *HIP* Hippocampus, *PAL* Lenticular nucleus, pallidum, *THA* Thalamus, *Cbe4-5* Cerebellum 4-5, *Cbe6* Cerebellum 6, *ORBsupmed* Superior frontal gyrus, medial orbital, *AMYG* Amygdala, *LING* Lingual gyrus, *PCL* Paracentral lobule, *STG* Superior temporal gyrus, *Cbe3* Cerebellum 3, *Cbe10* Cerebellum 10, *Ver1-2* Vermis 1-2, *Ver3* Vermis 3, *Ver4-5* Vermis 4-5, *Ver6* Vermis 6, *CEN* central executive network, *SMN* sensorimotor network, *DMN* default-mode network, *SN* salience network, *VN* visual network, *AN* auditory network

^*^Uncorrected* P* < 0.05; ^**^P_*FDR*_ < 0.05

### GHD-related subnetwork

**Fig. 4:**
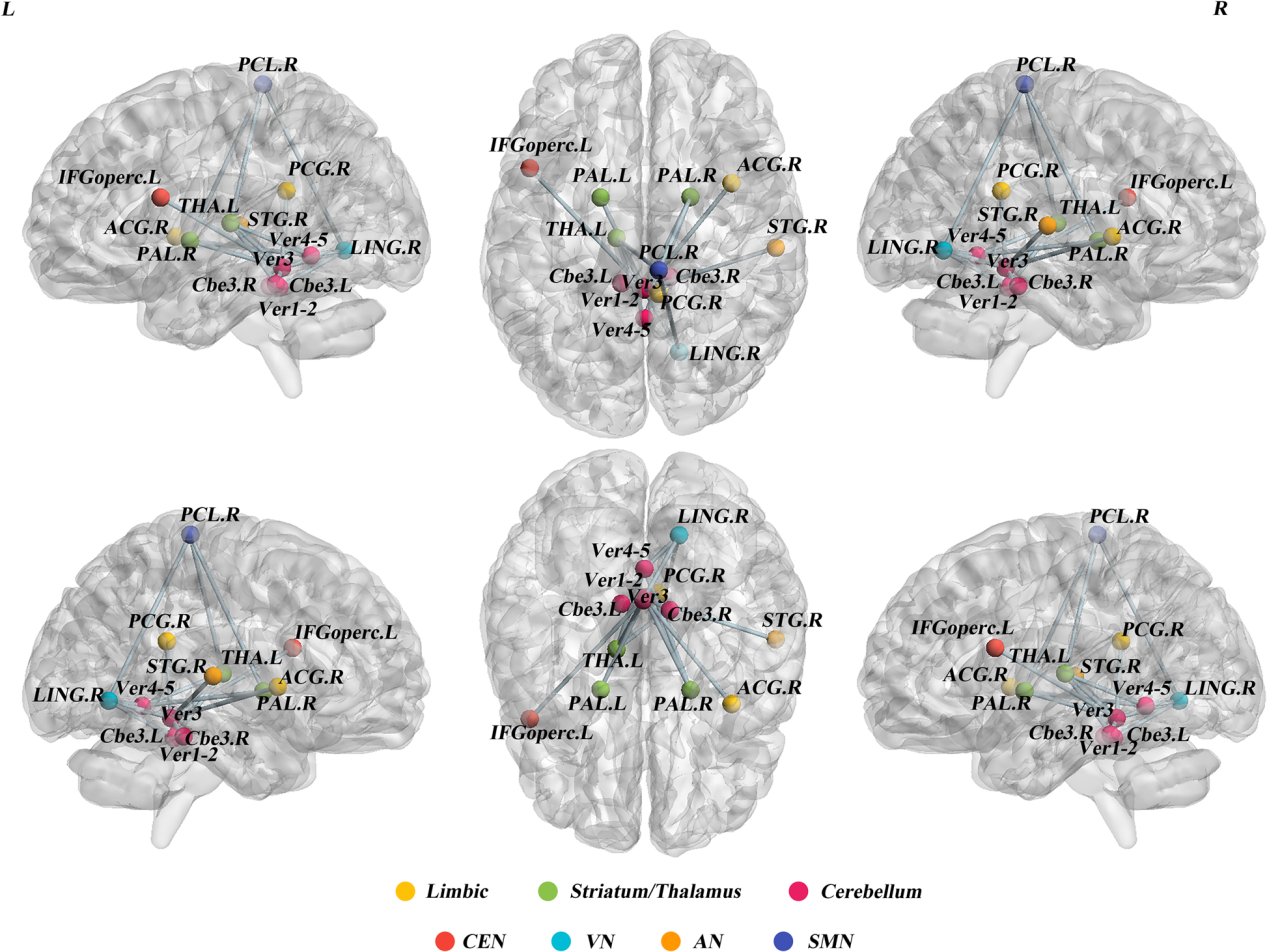
GHD-related subnetwork. Every node denotes a brain region, and every line represents a connection. Different-color nodes represent different brain regions: yellow, limbic regions; green, striatum/thalamus; pink, Cerebellum; red, central executive network (CEN); cyan, visual network (VN); orange, auditory network (AN); blue, sensorimotor network(SMN); Abbreviations: IFGoperc = Inferior frontal gyrus, opercular part; ACG = Anterior cingulate and paracingulate gyri; PCG = Posterior cingulate gyrus; PAL = Lenticular nucleus, pallidum; THA = Thalamus; LING = Lingual gyrus; PCL = Paracentral lobule; STG= Superior temporal gyrus; Cbe3 = Cerebellum 3; Ver1-2 = Vermis 1-2; Ver3 = Vermis 3; Ver4-5 = Vermis 4-5; L = left; R = right

For GHD patients, NBS analysis identified a significantly altered subnetwork with fourteen nodes and eighteen edges (Fig. 4). The nodes included components of cortico-striatal-thalamo-cerebellum loops and cortico-limbic-cerebellum network (paracentral-pallidum-vermis 3, 4). Additionally, dorsal visual systems-sensorimotor-striatal circuitry (lingual gyrus-paracentral lobule-pallidum) and auditory-cerebellum circuitry (superior temporal gyrus-vermis 3) were also found in GHD.

### Relationships between nodal profiles and serum markers and behavior scores

**Fig. 5:**
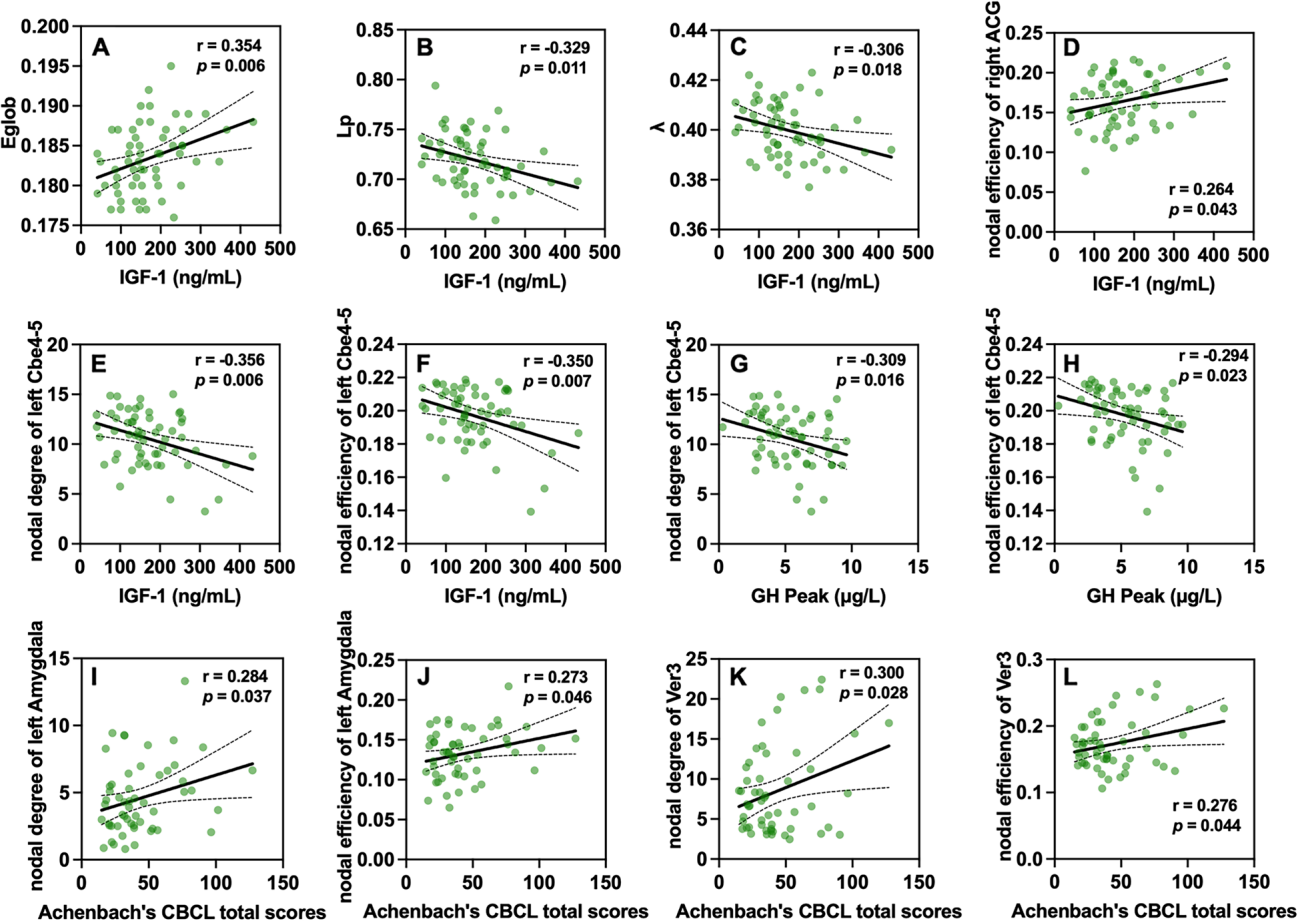
Relationship between the nodal properties and clinical variables in GHD group. In pediatric GHD, serum IGF-1 concentration was positively correlated with E_glob_ (*r* = 0.354; *P* = 0.006, **A**), and negatively correlated with L_p_ (*r* = -0.329; *P* = 0.011, **B**), λ (*r* = -0.306; *P* = 0.018, **C**). As for nodal profiles, serum IGF-1 concentration was positively correlated with nodal efficiency of the right ACG (*r* = 0.264; *P*= 0.043, **D**), and negatively correlated with nodal degree of the left cerebellum 4-5 (*r* = -0.356; *P* = 0.006, **E**), nodal efficiency of the left cerebellum 4-5 (*r* = -0.350; *P* = 0.007, **F**). Then, serum GH peak level was negatively correlated with nodal degree of the left cerebellum 4-5 (*r* = -0.309; *P* = 0.016, **G**) and nodal efficiency of the left cerebellum 4-5 (*r* = -0.294; *P* = 0.023, **H**). Total scores of Achenbach's CBCL were positively correlated with nodal degree of the left amygdala (*r* = 0.284; *P* = 0.037, **I**), nodal efficiency of the left amygdala (*r* = 0.273; *P* = 0.046, **J**), nodal degree of the vermis 3 (*r* = 0.300; *P* = 0.028, **K**), and nodal efficiency of the vermis 3 (*r* = 0.276; *P* = 0.044, **L**). Abbreviations: GHD = growth hormone deficiency; IGF-1 = insulin-like growth factor-1; ACG = anterior cingulate and paracingulate gyri

For whole-brain organization level (Fig. 5 A, B, C), serum IGF-1 concentration of GHD patients were positively correlated with E_glob_ (*r* = 0.354; *P* = 0.006, uncorrected), and negatively correlated with L_p_ (*r* = -0.329; *P* = 0.011, uncorrected), λ (*r* = -0.306; *P* = 0.018, uncorrected). As for nodal profiles (Fig. 5 D, E, F, G, H), in GHD patients, serum IGF-1 concentration was positively correlated with nodal efficiency of the right anterior cingulate and paracingulate gyri (*r* = 0.264; *P*= 0.043, uncorrected), and negatively correlated with nodal degree of the left cerebellum 4-5 (*r* = -0.356; *P* = 0.006, uncorrected), nodal efficiency of the left cerebellum 4-5 (*r* = -0.350; *P* = 0.007, uncorrected). Then, serum GH peak level was negatively correlated with nodal degree of the left cerebellum 4-5 (*r* = -0.309; *P* = 0.016, uncorrected) and nodal efficiency of the left cerebellum 4-5 (*r* = -0.294; *P* = 0.023, uncorrected).

As for behavior problem (Fig. 5 I, J, K, L), total scores of Achenbach’s CBCL were positively correlated with nodal degree of the left amygdala (*r* = 0.284; *P* = 0.037, uncorrected), nodal efficiency of the left amygdala (*r* = 0.273; *P* = 0.046, uncorrected), nodal degree of the vermis 3 (*r* = 0.300; *P* = 0.028, uncorrected), and nodal efficiency of the vermis 3 (*r* = 0.276; *P* = 0.044, uncorrected). No significant correlations were found between clinical variables and any other global or nodal metrics (*P* > 0.05). For the relationships between nodal profiles and serum markers and behavior scores, we report uncorrected *p*-values. Given the exploratory nature of these correlations, we considered *P* < 0.05 as significant, but these results should be interpreted with caution due to the multiple comparisons performed.

### Reproducibility of results

The reconstruction of the GM network using the HOA-112 atlas was repeated. Similar results characterized by lower E_glob_/E_loc_ and increased L_p_ were found in GHD than in TDs (Figure S1). In addition, significant altered nodal properties were observed in the prefrontal, limbic, striatum/thalamus, and visual/auditory regions in GHD patients while compared to TDs (details in Tables S2 Supplementary Materials; Figure S2). Moreover, GHD patients had similar disease-related subnetwork regions, including cortico-striatal-thalamo loops, limbic-striatum circuitry, and dorsal visual-limbic-striatal circuitry (supracalcarine cortex, hippocampus/amygdala, pallidum) and auditory systems (planum polare/superior temporal gyrus, posterior division) alterations (Figure S3). However, due to HOA-112 atlas did not involve cerebellum regions, cerebellum alterations were not evaluated.

## Discussion

The main findings of our study are: (i) GHD demonstrated large-scale disrupted brain network topologies: decreased integration and segregation; (ii) GHD showed disrupted cortico-striatal-thalamo-cerebellum loops, cortico-limbic-cerebellum network, dorsal visual systems-sensorimotor-striatal circuitry, and auditory-cerebellum circuitry; (iii) serum IGF-1 concentration was responsible for E_glob_, L_p_, λ and nodal profiles of right anterior cingulate and paracingulate gyri, left cerebellum 4-5, while GH peak was responsible for nodal degree, efficiency of left cerebellum 4-5; (iv) nodal profiles of left amygdala, vermis 3 were positively corrected with the total scores of Achenbach's CBCL. Overall, individual network-level abnormalities extend the understanding of grey matter maturational effects in GHD, which provide new insights into the pathophysiology of GHD.

### Decreased integration and increased segregation at the global level in GHD

The increased L_p_, γ, λ, σ and decreased C_p_, lower E_glob_ in GHD, indicated decreased integration and increased segregation of structural brain networks [11] and a disturbance in the normal small-world organization, a shift to stronger small-worldness in the GHD group [23]. The altered graphic measures in GHD likely reflect disruptions in normal brain development due to GH deficiency. GH and IGF-1 play crucial roles in neurogenesis, synaptic plasticity, and myelination. The decreased integration (lower global efficiency and higher λ) and increased segregation (higher γ) we observed may result from altered white matter development and reduced long-range connectivity [11, 30, 31]. In current study, the serum IGF-1 concentration was positively correlated with E_glob_, L_p_, λ, which indicated that large-scale anatomical organization were affected by the GH/IGF-1 axis [3]. Our results reinforced the understanding of altered brain morphological network due to growth hormone deficiency.

### Disrupted cortico-striatal-thalamo-cerebellum loops, cortico-limbic-cerebellum, and auditory, visual circuitry in GHD

Our work confirms and highlights the disrupted prefrontal cortico-striatal-thalamo-cerebellum loops, dorsal visual-sensorimotor-striatal circuitry and auditory-cerebellum circuitry associated with GHD, which were recently assumed and partly supported Jing et al.'s research within dynamic brain network [14]. Jing et al [14] have identified the interference of deficiency of GH on dynamic brain networks in children with short stature, mainly concentrated in the CEN and cerebellar network, as well as internetwork mainly including CEN-AN, CEN-SMN, CEN-VN, and cerebellar-DMN, cerebellar-SMN, cerebellar-VN. Those dysfunctional brain network regions were almost consistent with our findings (Table 2 & Fig. 2). It is well known that GH plays an important role in the cerebral cortex and cerebellar Purkinje cells [32]. The dorsolateral and orbitofrontal prefrontal-striatal circuits project from the basal ganglia to the prefrontal cortex via the thalamus's ventroanterior and dorsomedial regions [33]. Disruption in this circuitry demonstrates a 'fronto-subcortical' profile with a pattern of deficits such as impaired set-shifting and impairment of spatial working memory. We speculate that disrupted cortico-striato-thalamo-cerebellum loops, as well as dorsal visual systems-sensorimotor-striatal circuitry and auditory-cerebellum circuitry are participating in brain network organization, which are responsible for behavior problem symptoms.

Another vital circuitry involved with GHD was the limbic system. The limbic system expresses more GH and IGF-1 receptors [3, 5], including amygdala, hippocampus and parahippocampal areas, which are more severely affected by the GH/IGF-1 axis. The limbic system [34] is widely connected with other brain structures (neocortex, thalamus, brain stem), exchanging information between the midbrain, diencephalon and neocortex. We considered that lower connectivity in the limbic regions (bilateral amygdala) and higher connectivity in the limbic regions (anterior cingulate and paracingulate gyri, posterior cingulate gyrus, and left hippocampus) with other brain structures responsible for behavior problem symptoms in GHD children. Recently, a preliminary study of longitudinal changes showed that subjects with GHD had a smaller mean volume of the right thalamus, bilateral hippocampus, and bilateral amygdala than the controls [35] at baseline. We speculate that limbic regions were “intermediary hub” underlying GHD by enhancing internal anatomical connectivity (like left hippocampus) in compensatory to decreased volume.

### Relationships between nodal profiles and serum markers and behavior scores

The serum IGF-1 concentration was responsible for E_glob_, L_p_, λ and nodal profiles in right anterior cingulate, and serum IGF-1/GH peak concentration was responsible for paracingulate gyri and left cerebellum 4-5. Although GH and IGF-1 receptors are expressed throughout human brain [3], the limbic and cerebellar regions appears to be more susceptible to the GH/IGF-1 axis. Amygdala is a vital part of the limbic system [34] and is crucial in a wide array of effective and motivation-related behaviors. The cerebellum is traditionally associated with motor coordination and balance, also plays a crucial role in various aspects of higher-order function and dysfunction [36]. In addition, decreased nodal efficiency, degree of the left amygdala, vermis 3 were positively correlated with total scores of Achenbach's CBCL (reflecting behavior problem symptoms), which was unexpected and warrants further investigation. It may reflect compensatory mechanisms, where increased local efficiency develops in response to broader network disruptions. However, this increased local efficiency might come at the cost of reduced global integration, potentially contributing to behavioral issues.

The negative correlation between nodal degree and GH Peak in regions like the left cerebellum 4-5 suggests that more severe GH deficiency is associated with reduced connectivity in these areas. This aligns with the known role of GH in cerebellar development and function, and may contribute to the motor and cognitive symptoms often seen in GHD. Previous study have revealed that cerebellar dysfunction is evident in several developmental disorders, including autism, attention deficit-hyperactivity disorder (ADHD), and developmental dyslexia [37].Our results further highlights cerebellum's significance in regulating motivational and emotional states may due to its influence on autonomic function [36].

Based on current findings, the limbic and cerebellar regions (specifically amygdala and vermis 3) may be the susceptible target of the GH/IGF-1 axis and responsible for GHD behavior problem symptoms.

### Validation analyses

Validation analyses showed that significantly decreased E_glob_ and C_p_, as well as significantly altered nodal properties in the prefrontal, limbic, striatum/ thalamus, were similarly observed using the HOA-112 atlas in GHD than in TDs. Meanwhile, GHD showed decreased nodal degree and efficiency in the bilateral thalamus and amygdala according to both the AAL-90 and HOA-112 atlas, which uncovered the early involvement of the bilateral thalamus and amygdala. In addition, the GHD-related subnetwork was similar to those obtained from the AAL-90 atlas, implying the robustness of these findings.

### Limitations

The current study has several limitations. First, GHD includes partial (peak GH level ranges from 5 to 10 μg/L) and complete GHD (peak GH level less than 5 μg/L), the number of children with partial and complete GHD was evenly distributed in our study, but the difference between subtypes was not analyzed. Second, this study is lack of assessment of general cognitive functioning (IQ) in our participants. Future studies should include such measures to better characterize cognitive differences between GHD and TD groups and to investigate relationships between cognitive function and brain network properties. Lastly, longitudinal studies with GH replacement therapy are needed to examine neurodevelopmental alterations to evaluate the large-scale brain network organization.

## Conclusion

In summary, our findings suggest that pediatric GHD undergo an extensive and significant reorganization in MBNs, probably due to abnormal cortico-striatal-thalamo-cerebellum loops, cortico-limbic-cerebellum, dorsal visual-sensorimotor-striatal, and auditory-cerebellum circuitry. Detailed knowledge of large-scale network reorganization has the potential to help researchers better understand the pathophysiological mechanisms of GHD. In particular, the limbic and cerebellar regions (specifically amygdala and vermis 3) may be the susceptible target of the GH/IGF-1 axis, which might result in their relatively slower development in motor, cognitive, and linguistic functional within behavior problem performance.

### Supplementary Information

Supplementary Material 1: Table S1. Cortical and subcortical regions of HOA-112 Atlas. Table S2. Altered nodal profiles in GHD and TDs. Figure S1. Comparison of global parameters of the brain anatomical networks between the GHD group and typically developing (TD) controls. Abbreviations: GHD = growth hormone deficiency; TD = typically developing; E_glob_, global efficiency; E_loc_, local efficiency; C_p_, clustering coefficient; L_p_, shortest path length; λ, normalized characteristic path length; γ, normalized clustering coefficient; δ = λ/γ, small-world characteristic. Error bars represent the standard deviation of the mean. ^*^P < 0.05, compared with TDs. Figure S2. Compared to TDs, GHD group showed regions of altered nodal profiles, showing increased (red) and decreased (green) points. The detailed information can be found in Table S2. Abbreviations: GHD = growth hormone deficiency; TD = typically developing; INS = Insular Cortex; HIP = Hippocampus; CGa = Cingulate Gyrus, anterior division; TFp = Temporal Fusiform Cortex, posterior division; SCLC = Supracalcarine Cortex; PT = Planum Temporale; FO = Frontal Operculum Cortex; Bst = bed nucleus of the stria terminalis central division; Pal= Pallidum; Tha = Thalamus; SMC= Juxtapositional Lobule Cortex (formerly Supplementary Motor Cortex); T1p = Superior Temporal Gyrus, posterior division; PP= Planum Polare; Amy = Amygdala; L = left; R = right. Figure S3. GHD-related subnetwork. Every node denotes a brain region, and every line represents a connection. Different-color nodes represent different brain regions: red, central executive network (CEN); yellow, Limbic; green, Striatum/Thalamus; cyan, visual network (VN); blue, somatosensory network (SMN); pink, auditory network (AN); HIP = Hippocampus; CGa = Cingulate Gyrus, anterior division; SCLC = Supracalcarine Cortex; FO = Frontal Operculum Cortex; Pall = Pallidum; Thal = Thalamus; SMC = Juxtapositional Lobule Cortex (formerly Supplementary Motor Cortex); T1p = Superior Temporal Gyrus, posterior division; PP = Planum Polare; Amy = Amygdala; L = left; R = right.

## Data Availability

Data generated or analyzed during the study are available from the corresponding author by request. The data are not publicly available due to privacy or ethical restrictions.

## Abbreviations

GHD: Growth hormone deficiency
TDs: Typically developing controls
GM: Grey matter
MBNs: Morphological brain networks
AUC: Area under the curve
E_glob_: Global efficiency
E_loc_: Local efficiency
L_p_: Characteristic path length
C_p_: Clustering coefficient
γ: Normalized clustering coefficient
λ: Normalized characteristic path length
σ: Small-world parameters
MRI: Magnetic resonance imaging
AAL: Anatomical Automatic Labeling
CEN: Central executive network
SMN: Sensorimotor network
VN: Visual network

## Data availability

No datasets were generated or analysed during the current study.
