# Supplementary material for: Altered individual-level morphological similarity network in children with growth hormone deficiency
Source: J Neurodev Disord. 2024 Aug 26;16:48. doi: 10.1186/s11689-024-09566-5 (PMC11346214; doi:10.1186/s11689-024-09566-5)
Supplement: Supplementary file 1 — Supplementary Material 1: Table S1. Cortical and subcortical regions of HOA-112 Atlas. Table S2. Altered nodal profiles in GHD and TDs. Figure S1. Comparison of global parameters of the brain anatomical networks between the GHD group and typically developing (TD) controls. Abbreviations: GHD = growth hormone deficiency; TD = typically developing; Eglob, global efficiency; Eloc, local efficiency; Cp, clustering coefficient; Lp, shortest path length; λ, normalized characteristic path length; γ, normalized clustering coefficient; δ = λ/γ, small-world characteristic. Error bars represent the standard deviation of the mean. *P < 0.05, compared with TDs. Figure S2. Compared to TDs, GHD group showed regions of altered nodal profiles, showing increased (red) and decreased (green) points. The detailed information can be found in Table S2. Abbreviations: GHD = growth hormone deficiency; TD = typically developing; INS = Insular Cortex; HIP = Hippocampus; CGa = Cingulate Gyrus, anterior division; TFp = Temporal Fusiform Cortex, posterior division; SCLC = Supracalcarine Cortex; PT = Planum Temporale; FO = Frontal Operculum Cortex; Bst = bed nucleus of the stria terminalis central division; Pal= Pallidum; Tha = Thalamus; SMC= Juxtapositional Lobule Cortex (formerly Supplementary Motor Cortex); T1p = Superior Temporal Gyrus, posterior division; PP= Planum Polare; Amy = Amygdala; L = left; R = right. Figure S3. GHD-related subnetwork. Every node denotes a brain region, and every line represents a connection. Different-color nodes represent different brain regions: red, central executive network (CEN); yellow, Limbic; green, Striatum/Thalamus; cyan, visual network (VN); blue, somatosensory network (SMN); pink, auditory network (AN); HIP = Hippocampus; CGa = Cingulate Gyrus, anterior division; SCLC = Supracalcarine Cortex; FO = Frontal Operculum Cortex; Pall = Pallidum; Thal = Thalamus; SMC = Juxtapositional Lobule Cortex (formerly Supplementary Motor Cortex); T1p = Superior Temporal [file 11689_2024_9566_MOESM1_ESM.docx]

**Supplementary Materials**

**Table S1.** Cortical and subcortical regions of HOA-112 Atlas.

| Index | Region | Abbr. | Index | Region | Abbr. |
| --- | --- | --- | --- | --- | --- |
| (1,2) | Frontal Pole | FP | (57,58) | Cingulate Gyrus, anterior division | CGa |
| (3,4) | Insular Cortex | INS | (59,60) | Cingulate Gyrus, posterior division | CGp |
| (5,6) | Superior Frontal Gyrus | F1 | (61,62) | Precuneous Cortex | PCN |
| (7,8) | Middle Frontal Gyrus | F2 | (63,64) | Cuneal Cortex | CN |
| (9,10) | Inferior Frontal Gyrus, pars triangularis | F3t | (65,66) | Frontal Orbital Cortex | FOC |
| (11,12) | Inferior Frontal Gyrus, pars opercularis | F3o | (67,68) | Parahippocampal Gyrus, anterior division | PHa |
| (13,14) | Precentral Gyrus | PRG | (69,70) | Parahippocampal Gyrus, posterior division | PHp |
| (15,16) | Temporal Pole | TP | (71,72) | Lingual Gyrus | LG |
| (17,18) | Superior Temporal Gyrus, anterior division | T1a | (73,74) | Temporal Fusiform Cortex, anterior division | TFa |
| (19,20) | Superior Temporal Gyrus, posterior division | T1p | (75,76) | Temporal Fusiform Cortex, posterior division | TFp |
| (21,22) | Middle Temporal Gyrus, anterior division | T2a | (77,78) | Temporal Occipital Fusiform Cortex | TOF |
| (23,24) | Middle Temporal Gyrus, posterior division | T2p | (79,80) | Occipital Fusiform Gyrus | OF |
| (25,26) | Middle Temporal Gyrus, temporooccipital part | TO2 | (81,82) | Frontal Operculum Cortex | FO |
| (27,28) | Inferior Temporal Gyrus, anterior division | T3a | (83,84) | Central Opercular Cortex | CO |
| (29,30) | Inferior Temporal Gyrus, posterior division | T3p | (85,86) | Parietal Operculum Cortex | PO |
| (31,32) | Inferior Temporal Gyrus, temporooccipital part | TO3 | (87,88) | Planum Polare | PP |
| (33,34) | Postcentral Gyrus | POG | (89,90) | Heschl's Gyrus (includes H1 and H2) | H |
| (35,36) | Superior Parietal Lobule | SPL | (91,92) | Planum Temporale | PT |
| (37,38) | Supramarginal Gyrus, anterior division | SGa | (93,94) | Supracalcarine Cortex | SCLC |
| (39,40) | Supramarginal Gyrus, posterior division | SGp | (95,96) | Occipital Pole | OP |
| (41,42) | Angular Gyrus | AG | (97,98) | bed nucleus of the stria terminalis central division | Bst |
| (43,44) | Lateral Occipital Cortex, superior division | OLs | (99,100) | Thalamus | Thal |
| (45,46) | Lateral Occipital Cortex, inferior division | OLi | (101,102) | Caudate | Caud |
| (47,48) | Intracalcarine Cortex | CALC | (103,104) | Putamen | Put |
| (49,50) | Frontal Medial Cortex | FMC | (105,106) | Pallidum | Pall |
| (51,52) | Juxtapositional Lobule Cortex (formerly Supplementary Motor Cortex) | SMC | (107,108) | Hippocampus | Hip |
| (53,54) | Subcallosal Cortex | SC | (109,110) | Amygdala | Amy |
| (55,56) | Paracingulate Gyrus | PAC | (111,112) | Accumbens | Accbns |

Note: The regions are listed according to a prior template obtained from an HOA atlas; the odd number represents the corresponding brain regions in the left hemisphere; the even number denotes the specific brain regions in the right hemisphere.

**Table S2.** Altered nodal profiles in GHD and typically developing controls.

| **Brain regions** | **Category** | ***P*-value** | | |
| --- | --- | --- | --- | --- |
|  |  | $D_{i}^{auc}$ | $E_{i}^{auc}$ | $B_{i}^{auc}$ |
| **GHD > TDs** | | | | |
| INS.L | Limbic | 0.025* | 0.014* | 0.892 |
| HIP.L | Limbic | 0.018* | 0.016* | 0.981 |
| CGa.R | Limbic | 0.035* | 0.024* | 0.475 |
| TFp.L | VN | 0.009* | 0.033** | 0.146 |
| SCLC.L | VN | 0.080 | 0.048* | 0.042* |
| PT.R | AN | 0.028* | 0.013* | 0.845 |
| FO.L | CEN | 0.003* | 0.033** | 0.297 |
| Bst.L | Striatum | 0.023** | 0.033** | 0.753 |
| Pall.L | Striatum | 0.011* | 0.026 | 0.173 |
| Pall.R | Striatum | 0.205 | 0.013* | 0.003** |
| Thal.L | Thalamus | 0.023** | 0.033** | 0.464 |
| Thal.R | Thalamus | 0.015* | 0.004* | 0.375 |
| **GHD < TDs** | | | | |
| SMC.L | SMN | 0.031* | 0.027* | 0.432 |
| T1p.R | AN | 0.016* | 0.023* | 0.815 |
| PP.L | AN | 0.006** | 0.021** | 0.037* |
| Bst.R | Striatum | 0.638 | 0.001** | 0.005* |
| Amy.R | Limbic | 0.012* | 0.006* | 0.243 |

**Note:** 17 regions with *P*-value <0.05 in at least one node profile were included. * Uncorrected *P* < 0.05; ***P_FDR_* < 0.05. Abbreviations: $D_{i}^{auc}$= nodal degree; $E_{i}^{auc}$= nodal efficiency; $B_{i}^{auc}$= nodal betweenness; GHD = growth hormone deficiency; TD = typically developing; INS = Insular Cortex; HIP = Hippocampus; CGa = Cingulate Gyrus, anterior division; TFp = Temporal Fusiform Cortex, posterior division; SCLC = Supracalcarine Cortex; PT = Planum Temporale; FO = Frontal Operculum Cortex; Bst = bed nucleus of the stria terminalis central division; Pall = Pallidum; Thal = Thalamus; SMC = Juxtapositional Lobule Cortex (formerly Supplementary Motor Cortex); T1p = Superior Temporal Gyrus, posterior division; PP = Planum Polare; Amy = Amygdala; L = left; R = right; CEN = central executive network; VN = visual network; AN = auditory network.

**Figure Legends**


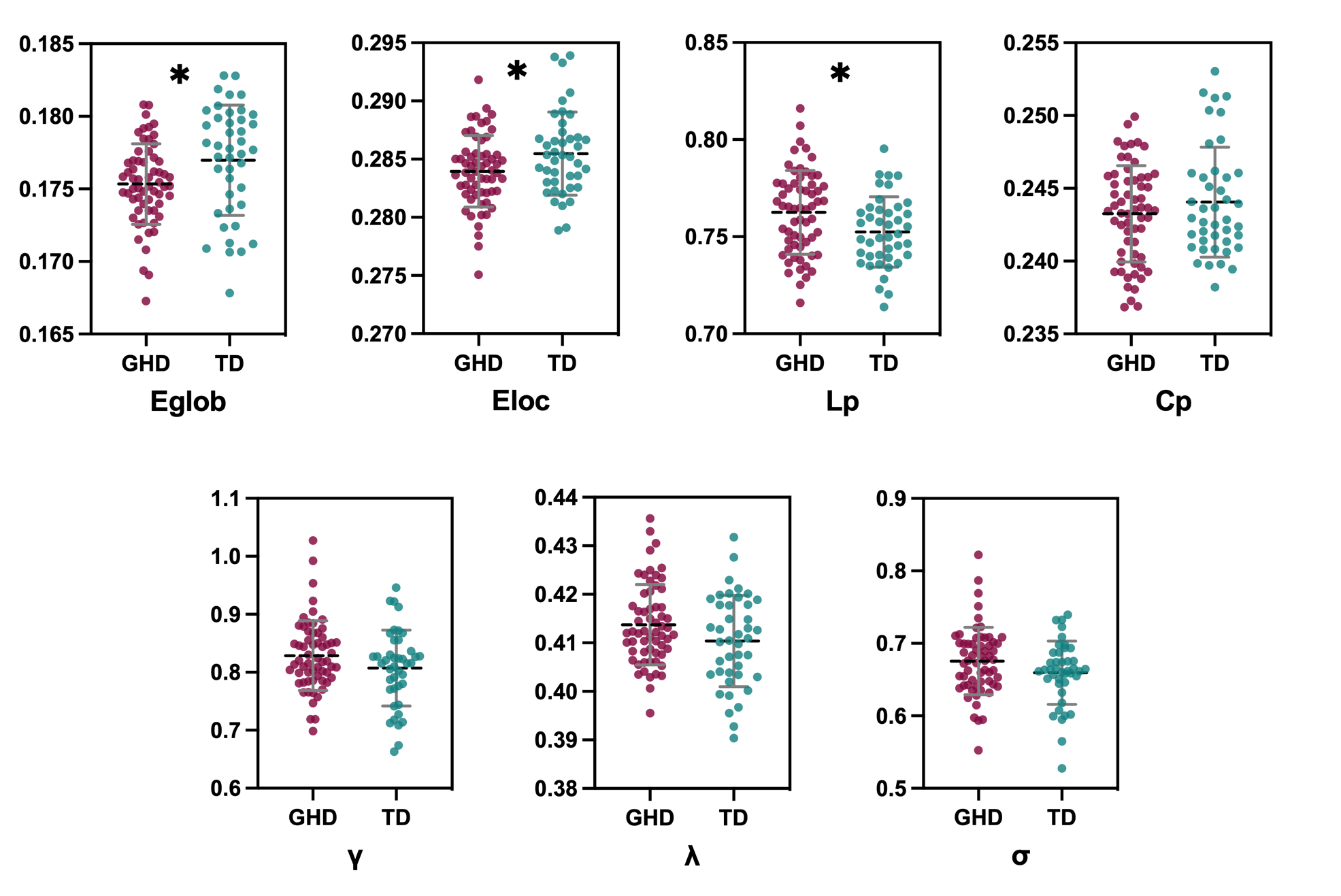


**Figure S1. Comparison of global parameters of the brain anatomical networks between the GHD group and typically developing (TD) controls.** Abbreviations: GHD = growth hormone deficiency; TD = typically developing; Eglob, global efficiency; Eloc, local efficiency; Cp, clustering coefficient; Lp, shortest path length; λ, normalized characteristic path length; γ, normalized clustering coefficient; δ = λ/γ, small-world characteristic. Error bars represent the standard deviation of the mean. **P* < 0.05, compared with TDs.

**
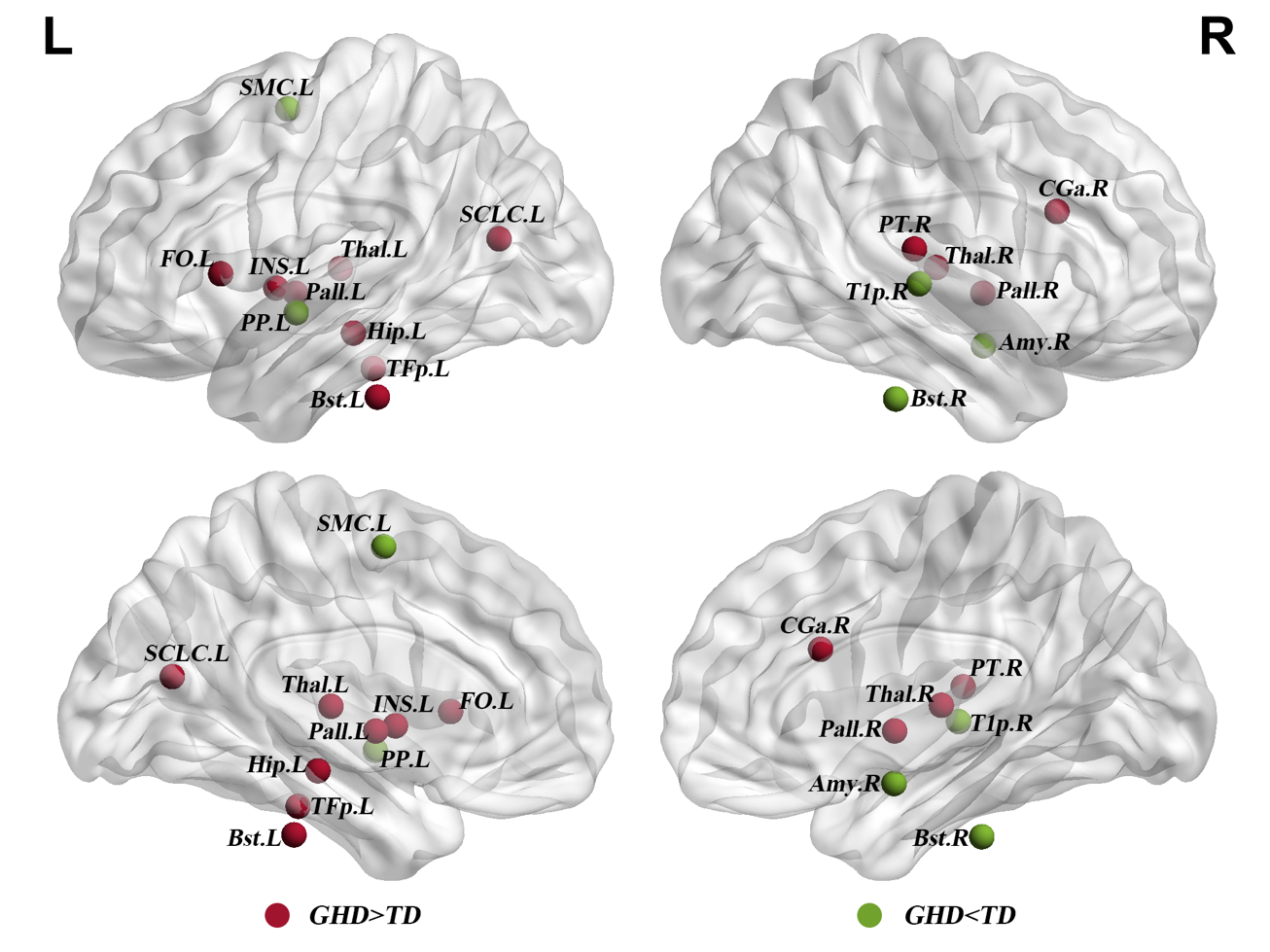
**

**Figure S2. Compared to TDs, GHD group showed regions of altered nodal profiles, showing increased points (red) and decreased (green) points.** The detailed information can be found in **Table S2**. Abbreviations: GHD = growth hormone deficiency; TD = typically developing; INS = Insular Cortex; HIP = Hippocampus; CGa = Cingulate Gyrus, anterior division; TFp = Temporal Fusiform Cortex, posterior division; SCLC = Supracalcarine Cortex; PT = Planum Temporale; FO = Frontal Operculum Cortex; Bst = bed nucleus of the stria terminalis central division; Pall = Pallidum; Thal = Thalamus; SMC = Juxtapositional Lobule Cortex (formerly Supplementary Motor Cortex); T1p = Superior Temporal Gyrus, posterior division; PP = Planum Polare; Amy = Amygdala; L = left; R = right.


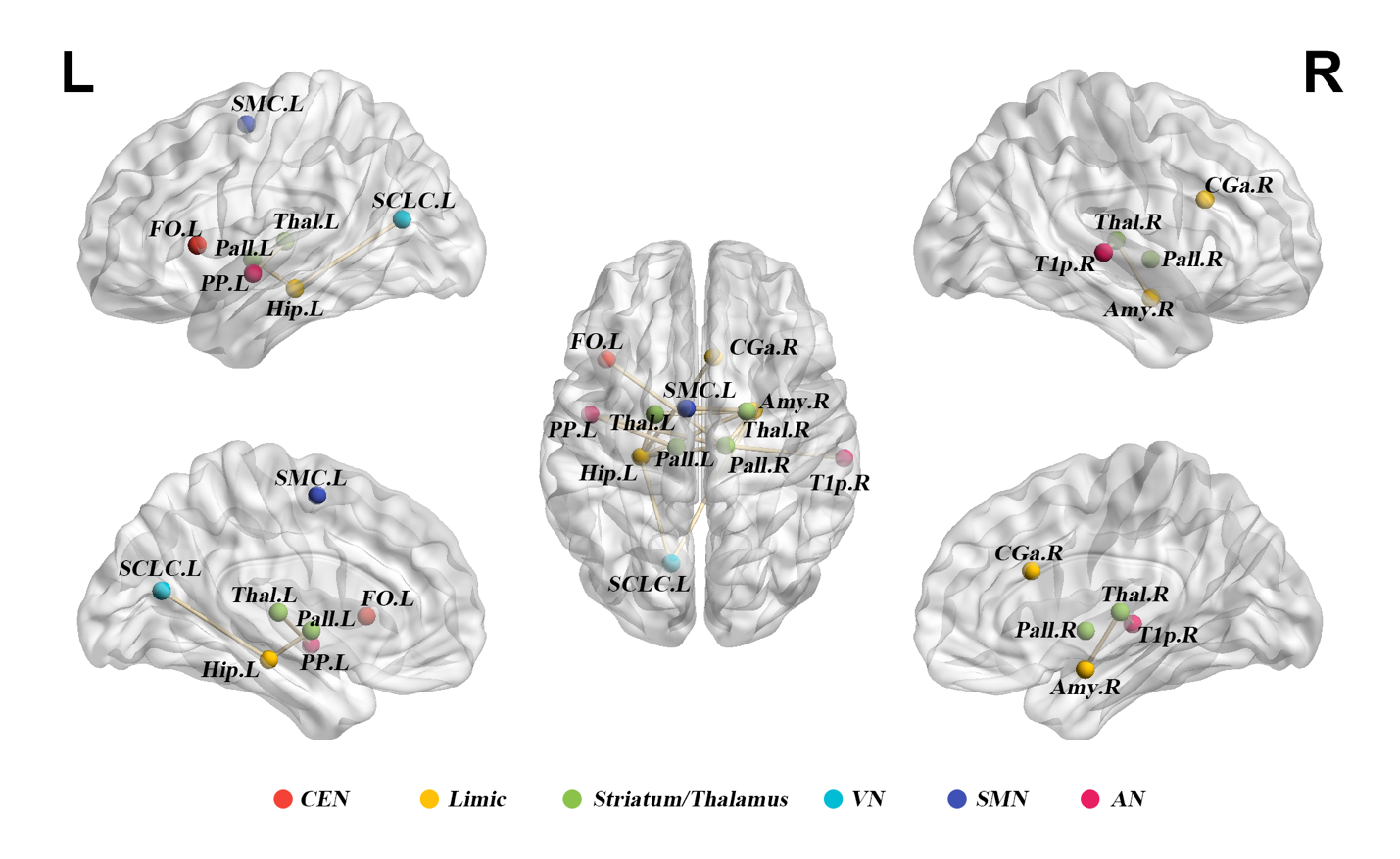


**Figure S3**. **GHD-related subnetwork.** Every node denotes a brain region, and every line represents a connection. Different-color nodes represent different brain regions: red, central executive network (CEN); yellow, Limbic; green, Striatum/Thalamus; cyan, visual network (VN); blue, somatosensory network (SMN); pink, auditory network (AN); HIP = Hippocampus; CGa = Cingulate Gyrus, anterior division; SCLC = Supracalcarine Cortex; FO = Frontal Operculum Cortex; Pall = Pallidum; Thal = Thalamus; SMC = Juxtapositional Lobule Cortex (formerly Supplementary Motor Cortex); T1p = Superior Temporal Gyrus, posterior division; PP = Planum Polare; Amy = Amygdala; L = left; R = right.
